# Supplementary material for: Thylacinus (Marsupialia: Thylacinidae) from the Mio-Pliocene boundary and the diversity of Late Neogene thylacinids in Australia
Source: PeerJ. 2015 May 12;3:e931. doi: 10.7717/peerj.931 (PMC4435473; doi:10.7717/peerj.931)
Supplement: Appendix S1 — The character list is provided in Yates (2014). [file peerj-03-931-s001.doc]

**Appendix 1. Character-Taxon Matrix**

The character list is provided in Yates (2014).

*Barinya wangala*

0 ? 0 0 0 0 0 1 0 0 0 0 0 0 0 0 0 0 0 0 0 0 0 1 0 0 0 0 0 0 0 0 0 0 0 0 0 0 0 0 0 0

*Antechinus flavipes*

0 0 0 0 0 1 0 0 0 0 0 0 0 0 0 0 0 0 0 0 0 0 0 0 0 0 0 0 1 0 0 0 0 0 0 0 0 0 0 0 0 0

*Muribacinus gadiyuli*

0 1 ? ? ? ? ? ? 1 0 0 0 0 0 0 0 0 0 0 0 0 0 0 0 1 0 0 0 ? 1 0 0 0 0 0 0 0 0 0 0 ? 0

*Badjcinus turnbulli*

0 0 ? 0 1 0 0 0 ? 0 0 ? 0 ? 0 0 0 0 0 0 ? ? 1 0 0 0 0 0 0 0 1 ? 2 0 1 0 1 0 0 0 1 0

*Mutpuracinus archibaldi*

0 ? 0 ? 0 0 0 0 1 0 0 0 1 0 0 0 0 0 0 0 0 0 0 1 0 0 0 0 0 0 1 ? ? 0 1 0 0 0 1 0 0 0

*Nimbacinus dicksoni*

0 0 0 1 1 0 0 0 1 0 0 0 1 ? 0 0 0 0 0 0 ? ? 0 1 0 ? 0 0 0 1 ? ? 1 0 1 0 0 0 0 0 0 0

*Nimbacinus richi*

? 0 0 ? ? 0 0 0 1 0 0 0 1 0 ? 0 0 0 1 0 0 0 0 1 0 0 0 0 1 1 1 ? 1 0 1 0 0 0 ? 0 0 0

*Maximucinus muirheadae*

? ? ? ? ? ? ? ? ? ? ? ? ? 1 0 ? 0 2 0 0 ? ? ? ? 1 0 ? ? ? ? ? ? ? ? ? ? ? ? ? ? ? 1

*Ngamalacinus timmulvaneyi*

? 1 ? ? ? 1 0 0 1 ? 0 0 ? ? 0 ? 0 0 0 0 0 ? 0 0 0 ? 1 0 ? ? ? ? 1 0 1 1 1 0 0 0 1 0

*Wabulacinus ridei*

0 0 ? ? ? ? ? ? ? 1 ? 1 1 ? 0 2 1 1 1 1 ? ? 1 0 1 1 1 ? ? 0 1 ? ? 1 1 0 0 1 ? 1 ? 0

*Tyarrpecinus rothi*

? ? ? ? ? 0 1 1 ? 1 0 0 1 0 1 1 0 1 0 1 ? ? 0 0 0 ? 0 0 ? ? ? ? ? ? ? ? ? 1 ? ? ? 0

*Thylacinus macknessi*

? ? ? ? ? ? ? ? ? 0 ? 1 1 ? 0 2 ? ? 1 0 ? ? 1 0 ? ? ? ? 0 1 1 0 2 1 1 1 0 1 0 1 1 0

*Thylacinus potens*

? ? 1 ? ? 0 1 1 1 0 0 1 ? 0 1 2 2 2 1 0 0 0 1 ? 1 0 0 0 1 0 0 0 2 2 2 1 0 1 1 1 0 2

*Thylacinus megiriani*

1 1 1 ? ? 0 0 1 ? 2 1 ? 0 0 1 ? 2 2 1 1 1 1 1 1 1 0 0 1 ? 1 1 1 ? 2 2 1 ? 1 ? ? 0 2

*Thylacinus cynocephalus*

2 1 0 1 1 1 0 0 0 2 1 1 1 1 1 2 2 2 1 1 1 1 1 0 1 2 1 1 0 1 1 1 2 2 2 1 0 1 1 1 0 1

*Thylacinus yorkellus*

? ? ? ? ? ? ? ? ? ? ? ? ? ? ? ? ? ? ? ? ? ? ? ? ? ? ? ? 0 1 1 1 2 2 2 1 0 1 ? 1 ? 1
